# The Five-DOF Explosion-Removal Manipulator Based on Position and Velocity Feedforward Compensation Control

**DOI:** 10.3390/s23094276

**Published:** 2023-04-25

**Authors:** Jianwei Zhao, Jiaxin Zhou

**Affiliations:** School of Mechanical Electronic & Information Engineering, China University of Mining and Technology (Beijing), Beijing 100083, China; zhaojianwei@cumtb.edu.cn

**Keywords:** five-degree-of-freedom collapsible manipulator, D-H model, forward/inverse kinematics analysis, position control, speed feedforward, Simulink simulation

## Abstract

The main problem with a robotic system arm is its sensitivity to time delays in the control process. Due to this problem, it is necessary to further optimize the control process of the system. One solution is to deal with the control accuracy and response speed issues of robotic arm joints, to improve the system’s response performance and enhance the system’s anti-interference ability. This paper proposes a speed feedforward and position control scheme for robotic arm joint control. The conclusion section shows that compared to traditional five-degree-of-freedom robotic arm systems, the addressed robotic arm control system has a lower tracking delay and better dynamic response performance. It can improve the system’s response performance while also enhancing its anti-interference ability.

## 1. Introduction

With the development and progress of social science and technology, robots are playing an increasingly important role in aerospace, medical equipment, explosion relief, and other fields [1,2]. An explosion-removal robot is a kind of robot that can take the place of people to investigate and deal with flammable and explosive dangerous goods in an extreme environment with an explosion risk. When the explosive disposal robot moves to the designated position, it carries out the operation task through the mechanical arm carried on the robot. The trajectory and holding position of the robot arm can be determined by visual observation within the visible range of human eyes. However, when the manipulator is in the over-the-line range, there will be a delay in remote image transmission, the robot arm joint control accuracy is not high, and the robot arm grasping task will have great interference [3]. Therefore, in the underlying control system of the robot arm, the accuracy and stability of joint control have always been the problems that need to be solved.

Gao et al. [4] designed a heavy-duty detonating robot that uses electric pushers as drive joints. The use of electric pushers is simple to control and allows for greater thrust. However, the robot arm is large in volume and the precision of the electric pusher control is not high. The robot arm produces large errors during the gripping process. The authors in [5] propose a nonlinear stability analysis of robot arm position control using a perturbation observer. The positional error can be reduced, and the dynamic response of the system can be improved, but the method needs to be further optimized in terms of robustness and noise sensitivity. Wang et al. [6] designed an adaptive slide robot control system with no sensory feedback. This strategy is effective for nonlinear precision control. However, the method requires a more accurate transmission model than adaptive slip mode control with sensory feedback in tracking performance. Wu et al. [7] proposed a reconfigurable robot force/position control method based on extended state observer constraints. Using Lyapunov theory to prove the stability of the system. This method can track and compensate for the target trajectory. However, the experiment only verified the position-tracking effect through simulation and did not conduct an actual test on the robotic arm device.

Based on the above research, this paper introduces a five-degree-of-freedom foldable manipulator. The manipulator is equipped with automated guided vehicles (AGVs) [8]. The AGVs control the chassis to move and work in complex terrain through remote commands. Then the explosive dangerous goods will be picked up and collected, and transported to the designated storage place. Due to the sensitivity of the five-degree-of-freedom robotic arm system to time-delay phenomena in the control process, further optimization of the system’s control process is needed. To address the control accuracy and response speed issues of robotic arm joints, to improve the system’s response performance while also enhancing the system’s anti-interference ability, this paper proposes a control scheme of the manipulator’s joints with velocity feedforward and position fusion [9]. A mathematical model of the robotic arm joint motor is established, and each joint motor adopts closed-loop feedback control of speed feedforward and position fusion. The joint motor loop uses a PID (Proportional Integral Derivative) controller, which can reduce the error and compensate for the uncertain disturbance. Finally, it is verified that the control accuracy of the robot arm joint can be guaranteed. At the same time, it can improve the rapid convergence of joint movement. The control system has a certain robustness to the variable load situation.

## 2. Five-Degree-of-Freedom Manipulator System

**Figure 1:**
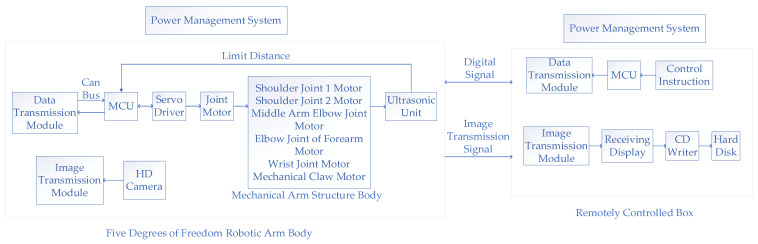
Five-degree-of-freedom manipulator system diagram.

The physical prototype used in this paper is a 5-DOF foldable manipulator. The whole manipulator system is composed of two parts, namely the ground station system and the main body of the five-degree-of-freedom manipulator. The system structure is shown in Figure 1. The main body of the 5-DOF manipulator consists of eight parts, including a power management unit, image transmission module, high-definition camera, data transmission module, embedded controller, motor driver, manipulator joint, and ultrasonic limit unit.

Its working process is that the high-definition camera collects the real-time video at the end of the manipulator, and transmits the video signal to the ground station through the image transmission module. After that, the data transmission module is used to receive the remote-control command sent by the ground station. The drive command to the servo driver is sent through the STM32 controller data processing, and the manipulator’s joint performs the corresponding action. To make the mechanical arm run to the specified position more accurately, four groups of ultrasonic modules are added for limit operation based on using an encoder for positioning.

### 2.1. Structural Design of Five-Degree-of-Freedom Foldable Manipulator

**Figure 2:**
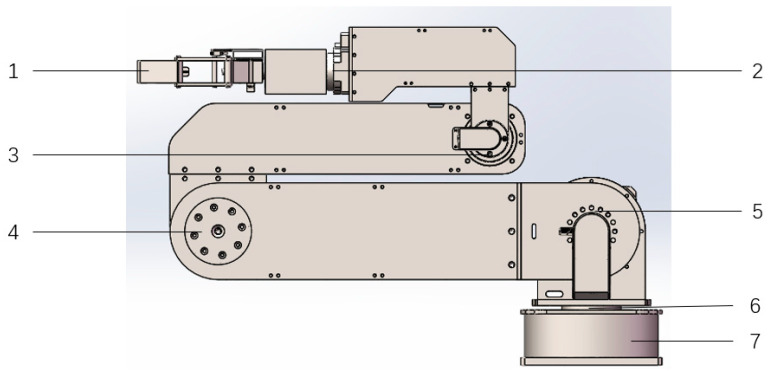
Structure diagram of mechanical arm folding state: 1. Mechanical Claw; 2. Wrist Joint; 3. Elbow Joint of Forearm; 4. Middle Arm Elbow; 5. Shoulder Joint 2; 6. Shoulder Joint 1; 7. Mechanical body.

The 5-DOF foldable manipulator consists of seven parts, namely the middle arm elbow, mechanical claw, wrist joint, elbow joint of the forearm, shoulder joint 1, shoulder joint 2, and mechanical body. The mechanical arm has a foldable function. When the mechanical arm is not working, the mechanical arm is in a folded state. When the mechanical arm is working, each joint is unfolded. The folding state structure of the manipulator is shown in Figure 2.

The limit range of the manipulator joint affects the workspace of the manipulator. According to the limit range of the joint, the trajectory of the manipulator can be better planned to make it reach the desired position reasonably. The range of motion of the manipulator joint is shown in Table 1.

**Figure 3:**
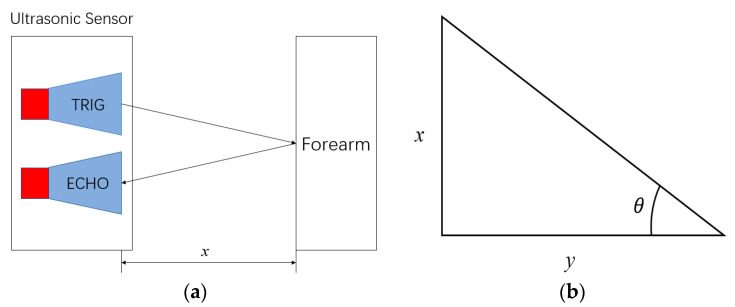
(**a**) Working principle of ultrasonic sensors; (**b**) Angle diagram of forearm elbow joint.

When the mechanical arm extends or retracts, it needs to accurately locate the position of the mechanical arm. Based on the control of the joint position of the mechanical arm, four external ultrasonic sensors are introduced to assist the positioning. The corresponding ultrasonic sensor information is collected through the stm32 controller and converted into the corresponding angle value of each joint [10]. Ultrasonic distance measurement adopts the echo detection method [11]. We take the forearm elbow joint as an example, as shown in Figure 3. The ultrasonic sensor has a sending part (TRIG) and a receiving part (ECHO). After the sending end sends the square wave to the forearm elbow joint (Forearm), the receiving end detects whether there is a signal return. *x* is the distance value of ultrasonic detection, *y* is the fixed position of ultrasonic on the middle arm, and *y* > 0, *θ* is the angle between the forearm and the middle arm, i.e., the required angle value. In the initial state of the manipulator, there is a relationship:

(1) $$tan\theta =\frac{x}{y}$$

From the inverse trigonometric function, the elbow joint angle of the forearm is:

(2) $$\theta =arctan\frac{x}{y}$$

We obtain the angle of the forearm elbow joint *θ*. After that, it is fused with the data fed back by the motor encoder to realize the redundant limit function.

### 2.2. Structural Design of Five-DOF Foldable Manipulator

**Figure 4:**
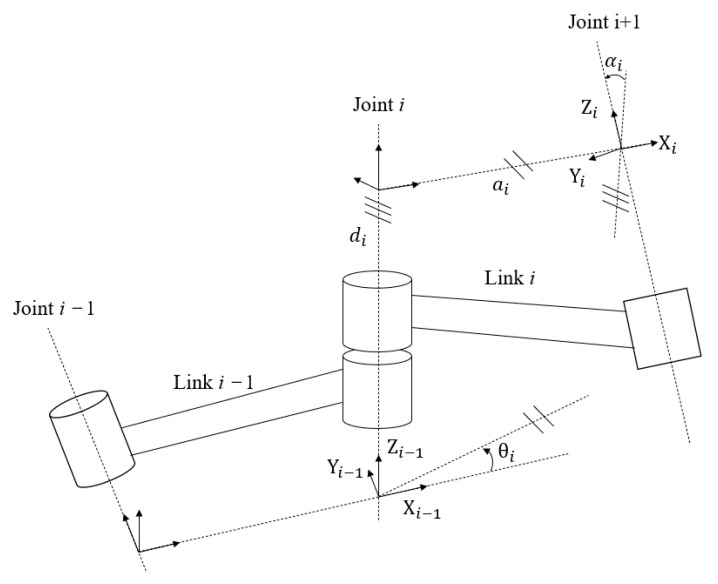
D-H method for establishing connecting rod coordinate system.

The D-H parameter method is a method that Denavit and Hartenberg proposed in 1955 to describe the rules of the kinematic relationship of the mechanism using the parameters of the connecting rod. In this paper, the standard D-H method is used to model the manipulator. Figure 4 shows the standard D-H linkage coordinate system [12].

D-H model modeling steps:

Around $Z_{i}$ axis rotation angle $\theta_{i}$. This is rotation transformation, which is represented by the rotation transformation operator [13]:

(3) $$Rot_{z_{i-1}}\left(\theta_{i}\right)=\left[\begin{array}{ccc} {\cos\theta }_{i} & -{\sin\theta }_{i} & 0\\ {\sin\theta }_{i} & {\cos\theta }_{i} & 0\\ 0 & 0 & 1 \end{array}\right]$$

From $X_{i-1}$ axis moves $d_{i}$ offset to $X_{i}$ axis. This is the translation transformation, which is represented by the translation transformation operator:

(4) $$Trans_{z_{i-1}}\left(d_{i}\right)=\left[\begin{array}{cccc} 1 & 0 & 0 & 0\\ 0 & 1 & 0 & 0\\ 0 & 0 & 1 & d_{i}\\ 0 & 0 & 0 & 1 \end{array}\right]$$

From $Z_{i-1}$ Axis moves the link length of $a_{i}$ to $Z_{i}$ axis. Transform for translation:

(5) $$Trans_{z_{i-1}}\left(a_{i}\right)=\left[\begin{array}{cccc} 1 & 0 & 0 & a_{i}\\ 0 & 1 & 0 & 0\\ 0 & 0 & 1 & 0\\ 0 & 0 & 0 & 1 \end{array}\right]$$

Around $X_{i}$ axis rotates clockwise the connecting rod angle $\alpha_{i}$, transform for rotation:

(6) $$Rot_{z_{i-1}}\left(\alpha_{i}\right)=\left[\begin{array}{ccc} 1 & 0 & 0\\ 0 & {\cos\alpha }_{i} & -{\sin\alpha }_{i}\\ 0 & {\sin\alpha }_{i} & {\cos\alpha }_{i} \end{array}\right]$$

Then the general transformation matrix from {*i*−1} coordinate system to {*i*} coordinate system is:

(7) Tii−1=cos⁡θi−sin⁡θicos⁡αisin⁡θisin⁡αiaicos⁡θisin⁡θicos⁡θicos⁡αi−cos⁡θisin⁡αiaisin⁡θi0sin⁡αicos⁡αidi0001

where Ti−1 represents the position and attitude of the *i*-th connecting rod relative to the *i*−1st connecting rod. The position and attitude of the end effector of the manipulator relative to the base coordinate system can be obtained by simply bringing in the D-H parameter of the connecting rod:

(8) Ti0=T10T21T32……Tii−1

**Figure 5:**
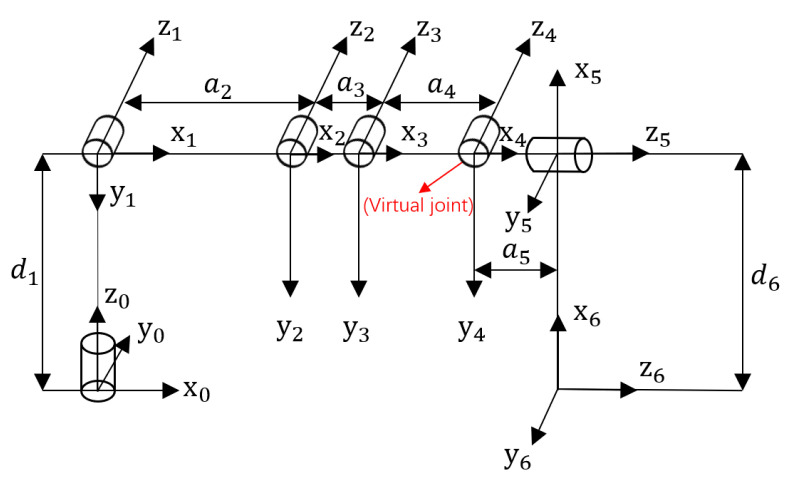
Structure diagram of the mechanical arm after introducing the virtual joint.

The manipulator designed in this paper is a manipulator with five degrees of freedom of rotation. Due to the complex structure of the manipulator, to simplify the modeling difficulty, a virtual joint can be established between two real joints [14]. Due to being a virtual joint, its angle value is fixed. After the introduction of the virtual joint, the manipulator has a total of six joints, of which the virtual joint is located between the elbow joint and the wrist joint of the forearm. The structure diagram of the virtual joint and the coordinate system established according to the standard D-H method are shown in Figure 5, the virtual joint is marked in the figure.

Based on the coordinate system of each joint established in Figure 4, the corresponding D-H parameter table can be obtained, as shown in Table 2. Joint angle $\theta_{i}$ in the table: the angle from $X_{i-1}$ to $X_{i}$ around axis $Z_{i-1}$; Offset distance $d_{i}$: the distance from $X_{i-1}$ to $X_{i}$ along axis $Z_{i-1}$; Connecting rod length $a_{i}$: the distance from $Z_{i-1}$ to $Z_{i}$ along axis $X_{i}$; Connecting rod torsion angle $\alpha_{i}$: the angle of rotation from $Z_{i-1}$ to $Z_{i}$ around axis $X_{i}$ [15].

### 2.3. Positive Kinematics Analysis of Manipulator

The forward kinematics analysis is to calculate the position and posture of the robot’s end under the condition that the robot’s joint variables, namely the joint angles $\theta_{i}$ known. According to the coordinate transformation rule of the manipulator, the homogeneous coordinate transformation matrix of each joint can be derived from Table 2 and Formula (7).

To express concisely, $sin\theta_{i}$ and $cos\theta_{i}$ is recorded as $S_{i}$ and $C_{i}$. Add $sin(\theta_{m}+\theta_{n})$, $cos(\theta_{m}+\theta_{n})$ and $\left(\theta_{m}+\theta_{n}\right)$ recorded as $S_{mn}$, $C_{mn}$ and $\theta_{mn}$, where *i*, *m*, *n* ∈ {0,1,2,3,4,5,6}.

(9) T10=C10S10S10−C10010d10001

(10) T21=C2−S20a2C2S2C20a2S200100001

(11) T32=C3−S30a3C3S3C30a3S300100001

(12) T43=C4−S40a4C4S4C40a4S400100001

(13) T54=C50S5a5C5S50−C5a5S501000001

(14) T65=C6−S600S6C600001d60001

We multiply the above formula to obtain the position and attitude of the center of the manipulator end actuator.

(15) T60=T10T21T32T43T54T65=nxoxqxpxnyoyqypynzozqzpz0001

In the formula:

$n_{x}=S_{1}S_{6}+C_{2345}C_{1}C_{6}$;

$n_{y}=C_{2345}C_{6}S_{1}-C_{1}S_{6}$;

$n_{z}=S_{2345}C_{6}$;

$o_{x}=C_{6}S_{1}-C_{2345}C_{1}S_{6}$;

$o_{y}=-C_{1}C_{6}-C_{2345}S_{1}S_{6}$;

$o_{z}=S_{2345}S_{1}$;

$q_{x}=S_{2345}C_{1}$;

$q_{y}=S_{2345}S_{1}$;

$q_{z}=-C_{2345}$;

$p_{x}=\left[a_{5}C_{2345}+d_{6}S_{2345}+a_{3}C_{23}+a_{2}C_{2}+a_{4}C_{234}\right]C_{1}$;

$p_{y}=\left[a_{5}C_{2345}+d_{6}S_{2345}+a_{3}C_{23}+a_{2}C_{2}+a_{4}C_{234}\right]S_{1}$;

$p_{z}=d_{1}-d_{6}C_{2345}+a_{5}S_{2345}+a_{3}S_{23}+a_{2}S_{2}+a_{4}S_{234}$;

The D-H parameters of the manipulator designed in this paper are known and at a given joint angle $\theta_{i}$, the pose matrix of the center point of the robot end actuator can be obtained through the above kinematics analysis.

### 2.4. Inverse Kinematics Analysis of Manipulator Based on Analytical Method

Inverse kinematics has a higher application value and is the basis of robot motion planning and trajectory planning [16]. There are usually two kinds of methods for solving inverse kinematics: the analytical method and numerical method. The analytical method is fast in operation, but has poor generality; the numerical method has high generality, but its solution speed is slow. By comparing the advantages and disadvantages of the two methods, the inverse kinematics analysis of the manipulator based on the analytical method is adopted in this paper. The sufficient condition for the manipulator to have the inverse kinematics analytical solution is to meet the Pieper criterion, i.e., to obtain the closed solution, one of two sufficient conditions needs to be satisfied [17,18,19].

(1) The three adjacent joint axes intersect at a point;
(2) The three adjacent joint axes are parallel to each other.

Shoulder joint 2, the middle arm elbow joint and the elbow joint of the forearm of the manipulator designed in this paper are parallel to each other, meeting the second sufficient condition, so the analytical solution can be obtained.

1. We solve joint angle $\theta_{1}$

We multiply both sides of Formula (15) by the matrix T10−1, the following equation can be obtained:

(16) T21T32T43T54T65=T10−1nxoxqxpxnyoyqypynzozqzpz0001

The following matrix can be obtained by simplifying the right side of Equation (16):

(17) $$\left[\begin{array}{cccc} n_{x}C_{1}+n_{y}S_{1} & o_{x}C_{1}+o_{y}S_{1} & q_{x}C_{1}+q_{y}S_{1} & p_{x}C_{1}+p_{y}S_{1}\\ n_{z} & o_{z} & q_{z} & p_{z}-d_{1}\\ n_{x}S_{1}-n_{y}C_{1} & o_{x}S_{1}-o_{y}C_{1} & q_{x}S_{1}-q_{y}C_{1} & p_{x}S_{1}-p_{y}C_{1}\\ 0 & 0 & 0 & 1 \end{array}\right]$$

The following matrix can be obtained by simplifying the left side of Equation (16):

(18) $$\begin{array}{cccc} C_{2345}C_{6} & S_{2345} & C_{2345}S_{6} & a_{5}C_{2345}+d_{6}S_{2345}+a_{3}C_{23}+a_{2}C_{2}+a_{4}C_{234}\\ S_{2345}C_{6} & -C_{2345} & S_{2345}S_{6} & a_{5}S_{2345}+d_{6}C_{2345}+a_{3}S_{23}+a_{2}S_{2}+a_{4}S_{234}\\ S_{6} & C_{6} & 0 & 0\\ 0 & 0 & 0 & 1 \end{array}$$

We observe the matrix (17) and (18), and select the element in the fourth column of the third row to obtain a $\theta_{1}$ and obtain the univariate linear Equation (19) [20],

(19) $$p_{x}S_{1}-p_{y}C_{1}=0$$

It can be solved as follows:

(20) $$\theta_{1}=atan2\left(p_{y},p_{x}\right)$$

2. We solve joint angle $\theta_{6}$

We continue to observe the matrix (17) and (18), and select the elements in the first and second columns of the third row to obtain a system of binary linear equations about $\theta_{6}$ (21),

(21) $$\left\{\begin{array}{c} n_{x}S_{1}-n_{y}C_{1}=S_{6}\\ o_{x}S_{1}-o_{y}C_{1}=C_{6} \end{array}\right.$$

Since $\theta_{1}$ is known, we can obtain:

(22) $$\theta_{6}=atan2\left(n_{x}S_{1}-n_{y}C_{1},o_{x}S_{1}-o_{y}C_{1}\right)$$

3. We solve joint angle $\theta_{2345}$

We continue to observe the matrix (17) and (18), and select the elements in the first and second rows of the second column to obtain a system of binary linear equations about $\theta_{2345}$ (23),

(23) $$\left\{\begin{array}{c} o_{x}C_{1}+o_{y}S_{1}=S_{2345}\\ o_{z}=-C_{2345} \end{array}\right.$$

Since $\theta_{1}$ is known, we can obtain:

(24) $$\theta_{2345}=atan2\left(o_{x}C_{1}+o_{y}S_{1},-o_{z}\right)$$

4. We solve joint angle $\theta_{4}$

Since $\theta_{4}$ is a virtual joint, so the joint angle value is fixed as:

(25) $$\theta_{4}=pi/2$$

5. We solve joint angle $\theta_{3}$

We continue to observe the matrix (17) and (18), and select the elements in the first and second rows of the fourth column to obtain a system of binary linear equations about $\theta_{23}$ and $\theta_{234}$ (26)

(26) $$\left\{\begin{array}{c} a_{5}C_{2345}+d_{6}S_{2345}+a_{3}C_{23}+a_{2}C_{2}+a_{4}C_{234}=p_{x}C_{1}+p_{y}S_{1}\\ a_{5}S_{2345}+d_{6}C_{2345}+a_{3}S_{23}+a_{2}S_{2}+a_{4}S_{234}=p_{z}-d_{1} \end{array}\right.$$

We move the term of equation group (26), move the known term to the left, and the unknown term to the right, and $\theta_{4}$ is known, so the trigonometric function identity transformation is used, and the equation set (27) can be obtained by simplifying the term of $\theta_{4}$:

(27) $$\left\{\begin{array}{c} p_{x}C_{1}+p_{y}S_{1}-a_{5}C_{2345}-d_{6}S_{2345}=a_{3}C_{23}+a_{2}C_{2}-a_{4}S_{23}\\ p_{z}-d_{1}-a_{5}S_{2345}+d_{6}C_{2345}=a_{3}S_{23}+a_{2}S_{2}+a_{4}C_{23} \end{array}\right.$$

We add the two sides of the equation set (4.25) after the square, which can be eliminated using the elimination method $\theta_{2}$. At the same time, using the triangular transformation formula, we can obtain:

(28) $$A^{2}+B^{2}-a_{2}^{2}-a_{3}^{2}-a_{4}^{2}=2a_{2}a_{3}C_{3}-2a_{2}a_{4}S_{3}$$

In the formula:

$$\begin{array}{c} A=p_{x}C_{1}+p_{y}S_{1}-a_{5}C_{2345}-d_{6}S_{2345};\\ B=p_{z}-d_{1}-a_{5}S_{2345}+d_{6}C_{2345} \end{array}$$

Using the trigonometric identity transformation, it is assumed that:

(29) $$\left\{\begin{array}{c} \eta \sin\varphi =a_{3}\\ \eta \cos\varphi =a_{4} \end{array}\right.$$

Then:

(30) $$\eta =\sqrt{a_{3}^{2}+a_{4}^{2}},\varphi =atan2\left(a_{3},a_{4}\right)$$

By introducing Equation (29) into Equation (28), we can obtain:

(31) $$\left\{\begin{array}{c} \sin\left(\varphi -\theta_{3}\right)=\frac{A^{2}+B^{2}-a_{2}^{2}-a_{3}^{2}-a_{4}^{2}}{2a_{2}\eta }\\ \cos\left(\varphi -\theta_{3}\right)=\pm \sqrt{1-{\left(\frac{A^{2}+B^{2}-a_{2}^{2}-a_{3}^{2}-a_{4}^{2}}{2a_{2}\eta }\right)}^{2}} \end{array}\right.$$

According to Equation (31):

(32) $$\begin{array}{l} \theta_{3}=atan2\left(a_{3},a_{4}\right)\\ \;\;\;\;\;\;-atan2(A^{2}+B^{2}-a_{2}^{2}-a_{3}^{2}\\ \;\;\;\;\;\;-a_{4}^{2},\pm \sqrt{\left[4a_{2}^{2}\left(a_{3}^{2}+a_{4}^{2}\right)-\left(A^{2}+B^{2}-a_{2}^{2}-a_{3}^{2}-a_{4}^{2}\right)\right]}) \end{array}$$

6. We solve joint angle $\theta_{5}$

So far, the values of $\theta_{1}$, $\theta_{3}$, $\theta_{4}$, $\theta_{6}$, $\theta_{2345}$ have been solved. We continue to observe the matrices (17) and (18), and the solution to obtain $\theta_{5}$ continues to simplify Equation (16) and multiply both sides of Equation (16) by T65−1, T54−1, and the matrix can be obtained by simplifying the left side of the equation:

(33) $$\left[\begin{array}{cccc} C_{234} & -S_{234} & 0 & a_{3}C_{23}+a_{2}C_{2}+a_{4}C_{234}\\ S_{234} & C_{234} & 0 & a_{3}S_{23}+a_{2}S_{2}+a_{4}S_{234}\\ 0 & 0 & 1 & 0\\ 0 & 0 & 0 & 1 \end{array}\right]$$

On the right side of the simplified Equation (16), we can obtain $\theta_{5}$. Because the matrix is too complex, only the second row of elements needed is listed here:

(34) $$\left[\begin{array}{cccc} q_{z}S_{5}+n_{z}C_{5}C_{6}-o_{z}C_{5}S_{6} & n_{z}C_{6}S_{5}+q_{z}C_{5}-o_{z}S_{5}S_{6} & o_{z}C_{6}+n_{z}S_{6} & p_{z}-d_{1}-q_{z}d_{6}-a_{5}n_{z}C_{6}+a_{5}o_{z}S_{6} \end{array}\right]$$

By observing the matrix (33) and (34), select the elements in the first and second columns of the second row to obtain the information about $\theta_{5}$.

(35) $$\left\{\begin{array}{c} q_{z}S_{5}+n_{z}C_{5}C_{6}-o_{z}C_{5}S_{6}=S_{234}\\ n_{z}C_{6}S_{5}+q_{z}C_{5}-o_{z}S_{5}S_{6}=C_{234} \end{array}\right.$$

At this point, the trigonometric function equation of $\theta_{234}$ and $\theta_{2345}$ are brought into the equation group (26), and the equation transformation is performed again to obtain the following formal equation group:

(36) $$\left\{\begin{array}{c} a_{2}C_{2}=P-\left(XS_{5}+YC_{5}\right)\\ a_{2}S_{2}=Q-\left(XC_{5}+YS_{5}\right) \end{array}\right.$$

In the formula:

$$\begin{array}{c} N=n_{z}C_{6}-o_{z}S_{6};\\ P=-(-a_{5}q_{z}+d_{6}\left(q_{x}C_{1}+q_{y}S_{1}\right)-p_{x}C_{1}-p_{y}S_{1});\\ Q=-\left(a_{5}\left(q_{x}C_{1}+q_{y}S_{1}\right)+d_{6}q_{z}-p_{z}+d_{1}\right);\\ X=a_{3}q_{z}+a_{4}N;\\ Y=a_{3}N-a_{4}q_{z} \end{array}$$

We use the elimination method to square and add the two sides of the equation group (36), which can be eliminated $\theta_{2}$ relevant elements, the following equation is obtained:

(37) $$2\left(QY-PX\right)S_{5}-2\left(QX+PY\right)C_{5}=a_{2}^{2}-P^{2}-Q^{2}-X^{2}-Y^{2}$$

Using the trigonometric identity transformation, it is assumed that:

(38) $$\left\{\begin{array}{c} \eta \sin\varphi =2\left(QY-PX\right)\\ \eta \cos\varphi =2\left(QX+PY\right) \end{array}\right.$$

Then:

(39) $$\eta =\sqrt{2{\left(QY-PX\right)}_{}^{2}+2{\left(QX+PY\right)}_{}^{2}}$$

(40) $$\varphi =atan2\left(2\left(QY-PX\right),2\left(QX+PY\right)\right)$$

By introducing Equation (38) into Equation (37), we can obtain:

(41) $$\begin{array}{l} \theta_{5}=atan2\left(\pm \sqrt{\left(M-{\left(P^{2}+Q^{2}+X^{2}+Y^{2}-a_{2}^{2}\right)}^{2}\right)},P^{2}+Q^{2}+X^{2}+Y^{2}-a_{2}^{2}\right)\\ \;\;\;\;\;\;\;\;-atan2\left(2\left(QY-PX\right),2\left(PY+QX\right)\right) \end{array}$$

In the formula:

$$\begin{array}{c} N=n_{z}C_{6}-o_{z}S_{6};\\ P=-(-a_{5}q_{z}+d_{6}\left(q_{x}C_{1}+q_{y}S_{1}\right)-p_{x}C_{1}-p_{y}S_{1});\\ Q=-\left(a_{5}\left(q_{x}C_{1}+q_{y}S_{1}\right)+d_{6}q_{z}-p_{z}+d_{1}\right);\\ X=a_{3}q_{z}+a_{4}N;\\ Y=a_{3}N-a_{4}q_{z};\\ M=4\left({\left(QY-PX\right)}^{2}+{\left(PY+QX\right)}^{2}\right) \end{array}$$

7. We solve joint angle $\theta_{2}$

According to the principle that joint angles can be vector added, we can obtain the joint angle of $\theta_{2}$:

(42) $$\theta_{2}=\theta_{2345}-\theta_{3}-\theta_{4}-\theta_{5}$$

We can obtain four joint combinations of the manipulator in the same position and posture based on Formulas (32) and (41). Combined with the rotatable angle range of each joint of the manipulator, when the target position and pose T of the end effector of the manipulator is given, the inverse kinematics solution is performed, and only one of the four joint combinations obtained meets the rotation value range of the joint.

## 3. Kinematics Simulation Verification of Manipulator

### 3.1. Simulation and Verification of Forward Kinematics Equation

**Figure 6:**
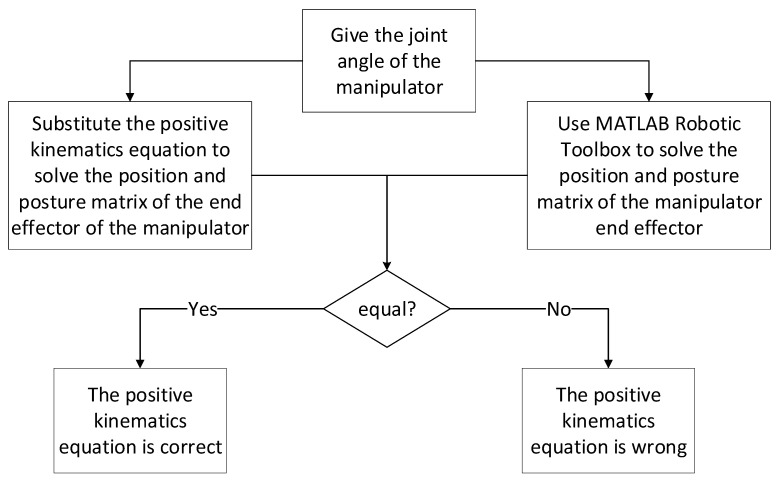
Flow chart of forward kinematics analysis and verification of 5-DOF folding manipulator.

The accuracy of the analysis must be verified by forward kinematics analysis after the forward kinematics equation of the 5-DOF foldable anti-explosion manipulator is obtained. The pose matrix of the end effector of the manipulator is obtained by substituting the given joint angles of the manipulator into the forward kinematics equation. The pose matrix is compared with the pose matrix obtained using the MATLAB Robotic Toolbox. If they are equal, the positive kinematics equation is correct. The verification process of forward kinematics analysis is shown in Figure 6.

The D-H parameter of the 5-DOF foldable manipulator obtained from the parameter table: the length of the connecting rod $a_{2}=0.5m$, $a_{3}=0.125m,a_{4}=0.366m,a_{5}=0.075m$; Joint offset $d_{1}=0.2m,d_{6}=0.3m$. At the same time, we change the initial joint angle $\theta_{i}$. (*i* = 1,2,3,5,6) is assigned to 0, i.e., $\theta_{i}=0{}^{\circ}$, (*i* = 1,2,3,5,6), the virtual joint angle value is fixed, $\theta_{4}=90{}^{\circ}$. We bring the above data into Equation (2) to obtain the position and attitude matrix of the manipulator end effector relative to the base coordinate system:

(43) T60=0010.9250−1001000.6410001

**Figure 7:**
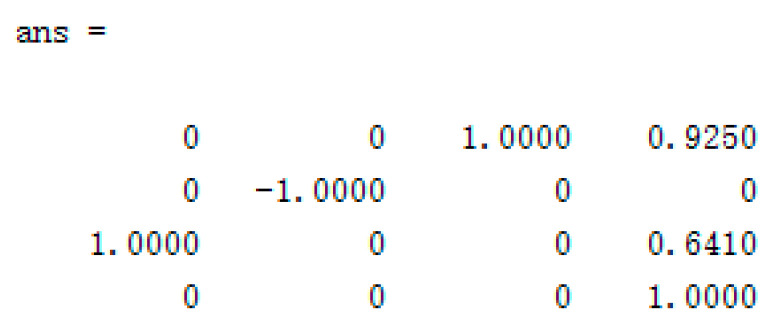
MATLAB Robotic Toolbox to solve forward kinematics calculation results.

Using the MATLAB Robotic Toolbox, it is convenient to obtain the position and attitude matrix of the manipulator end effector relative to the base coordinate system. Given the joint space matrix of manipulator $\theta =\left[\begin{array}{cccccc} 0 & 0 & 0 & P_{i}/2 & 0 & 0 \end{array}\right]$, we use the fkine forward kinematics solution function contained in the MATLAB Robotic Toolbox to obtain the position and posture matrix of the manipulator end effector, and the calculation results are shown in Figure 7.

**Figure 8:**
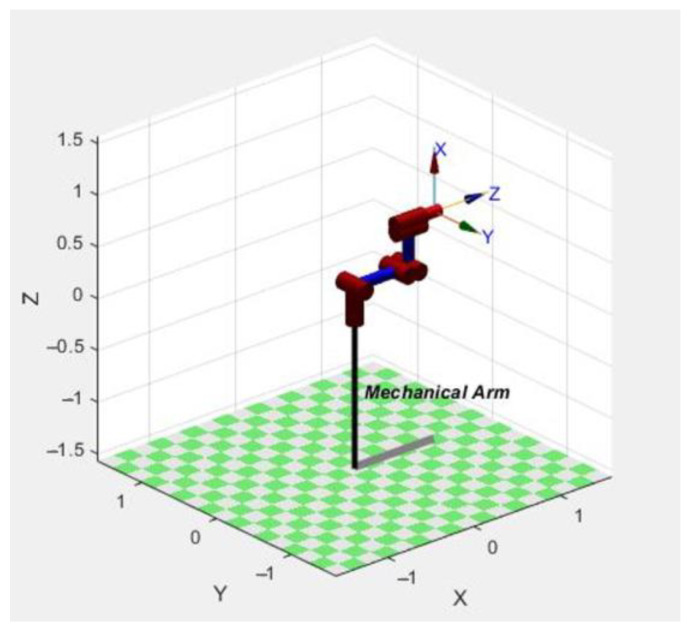
Kinematic model of the manipulator in the initial state.

Through comparison, the position and posture matrix of the end effector of the manipulator solved by the MATLAB Robotic Toolbox is completely consistent with the result obtained by Equation (3), which shows that the forward kinematics analysis result of the upper arm is correct. At the same time, the matrix of the manipulator in the manipulator joint space can be obtained when *θ* = [0,0,0, $P_{i}/2$,0,0] using the MATLAB Robotic Toolbox, as shown in Figure 8, where the coordinate units of the *X*, *Y*, and *Z* axes in this spatial matrix are all m.

### 3.2. Simulation and Verification of Inverse Kinematics Equation

**Figure 9:**
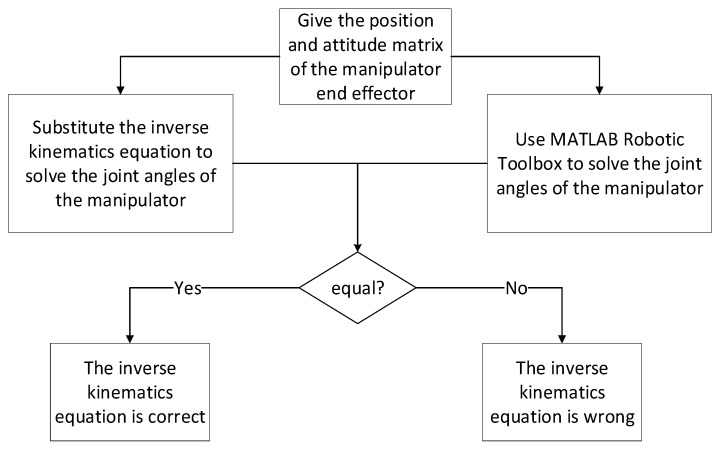
Flow chart of inverse kinematics analysis and verification of 5-DOF folding manipulator.

Through the inverse kinematics analysis, the accuracy of the analysis must be verified after the inverse kinematics equation of the 5-DOF foldable anti-explosion manipulator is obtained. Through the given pose matrix of the manipulator end effector, the angle of each joint of the manipulator is solved by substituting it into the inverse kinematics equation, and the angle is compared with the angle value obtained using the MATLAB Robot Toolbox. If they are equal, the inverse kinematics equation is correct. The validation process of inverse kinematics analysis is shown in Figure 9.

To prove the correctness of the inverse kinematics algorithm of the manipulator, the joint space matrix of the manipulator is given first *θ* = [$P_{i}/3$, $P_{i}/3$, −$P_{i}/3$, $P_{i}/2$, $P_{i}/6$, $P_{i}/2$]. The forward kinematics solution of the manipulator, namely the position and posture matrix of the manipulator end actuator is obtained through the forward kinematics algorithm of the manipulator, and it is substituted into the inverse kinematics equation to obtain four inverse kinematics solutions, as shown in Table 3. The second group of solutions and space matrix *θ* = [$P_{i}/3$, $P_{i}/3$, −$P_{i}/3$, $P_{i}/2$, $P_{i}/6$, $P_{i}/2$] are the same, which verifies the correctness of the inverse kinematics algorithm.

The forward and inverse kinematics analysis and simulation verification of the manipulator can provide the theoretical and data basis for the actual motion control of the manipulator. The spatial analysis simulation, trajectory planning, and simulation of the manipulator joint all need the theoretical support of forward and inverse kinematics analysis.

## 4. Ground Station Control System

**Figure 10:**
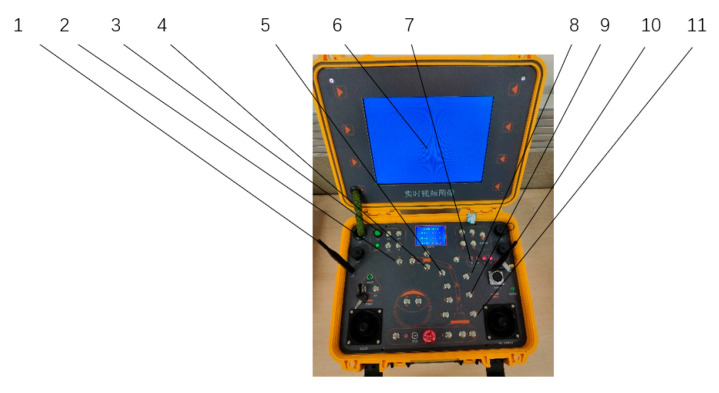
Ground station control system: 1. Data transmission antenna; 2. Button of mechanical claw; 3. Power switch; 4. Button of elbow joint; 5. Button of forearm elbow joint; 6. Display screen; 7. Power indicator; 8. Image transmission antenna; 9. Button of middle arm elbow joint; 10. Shoulder joint 2 button; 11. Shoulder joint 1 button.

The ground station control system consists of three parts, as shown in Figure 10, which are the power management system, video unit, and manipulator controller. The power management system provides the ground station system power supply voltage. The video unit receives the video signal through the image transmission module, uses the display to return the real-time picture, and saves the video to the mobile hard disk through the recorder. The manipulator controller interface is composed of a joint key, power switch, power indicator, data transmission antenna, image transmission antenna, and image display screen. The robot arm can be remotely controlled through the robot arm control box to carry out operation tasks, and the end image of the robot arm can be displayed in real time through the display screen.

## 5. Mechanical Arm Joint Composition and Mathematical Model Establishment

### 5.1. Parameters of Wrist Joint Motor

Traditional DC motors use brushes for mechanical commutation [21], so there is mechanical friction, which shortens the service life of the motor and presents problems such as noise, sparks, and radio interference. In addition, high manufacturing costs and difficult maintenance limit its application on some special occasions. A brushless DC motor has high efficiency, long life, low noise, and good speed-torque characteristics [22]. Considering the above factors, this paper selects a brushless DC motor as the joint drive motor of the manipulator. Because the five joint motors in the manipulator all use the same control method, the fifth-axis wrist joint is selected for analysis in this paper. The wrist joint motor parameters are shown in Table 2.

**Figure 11:**
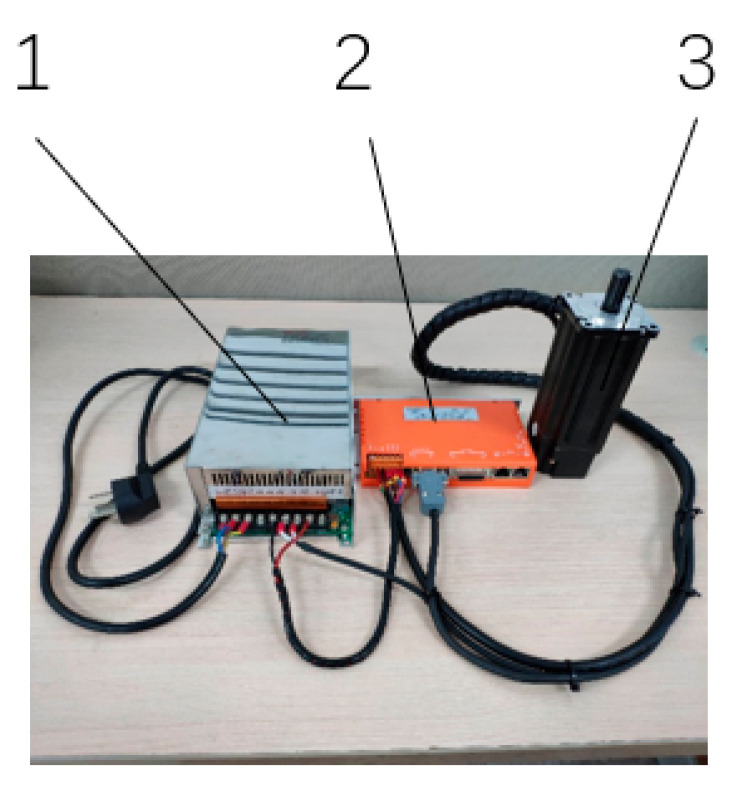
Wiring diagram of motor and driver: 1. Power supply; 2. Driver; 3. DC brushless motor.

**Figure 12:**
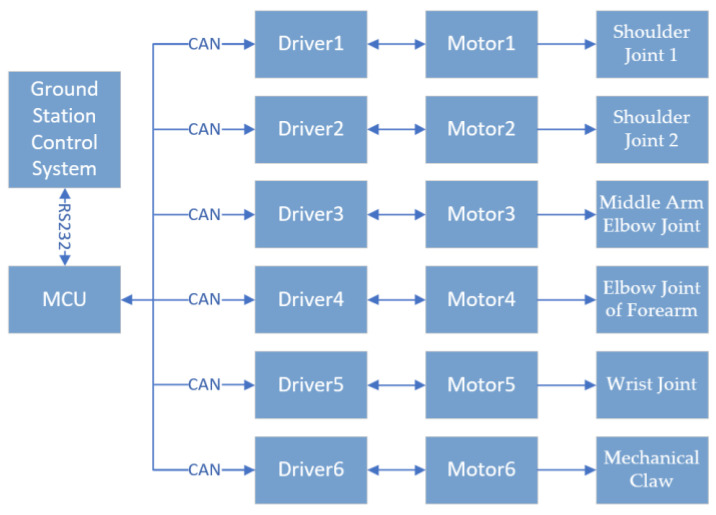
Overall control process of the robotic arm.

Figure 11 shows the wiring diagram of the power supply, driver, and DC brushless motor. The input voltage of the switching power supply is AC 220 V, and the output voltage is DC 48 V, providing the power supply voltage of the driver and servo motor. The controller communicates with the driver using RS232 protocol, and the motor is connected to the driver through a network cable. The CAN bus protocol is used for communication between the controller and driver [23]. The modular design requires only one network cable to connect, which simplifies the wiring steps and saves the working space. The overall control process of the robotic arm is shown in Figure 12. The relevant parameters and interface functions of the controller are shown in Table 4.

### 5.2. Establishment of Mathematical Model of Wrist Joint Motor (Constraint Conditions of Manipulator)

The mathematical equation model of the wrist joint motor is established. The voltage of any phase in the winding can be expressed as [24]:

(44) $$u_{x}=R_{x}i_{x}+e_{\psi x}$$

where $u_{x}$ refers to phase voltage, subscript *x* refers to three-phase winding *A*, *B*, *C*, $i_{x}$ refers to phase current, $e_{\psi x}$ is phase-induced potential, $R_{x}$ is phase resistance, $R_{a}=R_{b}=R_{c}=R$ in three-phase symmetrical winding.

(45) $$e_{\psi x}=\frac{d\psi x}{dt}$$

Because each phase winding and its current produces flux cross-chain, it also produces flux and permanent magnet cross-chain with other winding currents. Take phase A winding as an example:

(46) $$\psi_{A}=L_{A}i_{A}+M_{AB}i_{B}+M_{AC}i_{C}+\psi_{pm}\left(\theta \right)$$

In the formula, $\psi_{pm}(\theta )$ is the permanent magnetic linkage of the turning chain of phase *A* winding, θ is the rotor position angle, $L_{A}$ is self-induction of phase *A* winding; $M_{AB}$ and $M_{AC}$ are the mutual inductance of phase *B* winding and phase *C* winding to phase *A* winding.

Phase *A* voltage can be derived from Formula (44) to Formula (46) [25]:

(47) $$u_{A}={Ri}_{A}+\left(L-M\right)\frac{di_{A}}{dt}+e_{A}$$

After deduction, similar results are obtained for phases *B* and *C*. Since phase voltage is not easy to measure in practical applications, the mathematical model of line voltage is more in line with the actual situation, expressed as [26]:

(48) $$\left[\begin{array}{c} u_{AB}\\ u_{BC}\\ u_{CA} \end{array}\right]=\left[\begin{array}{ccc} R & -R & 0\\ 0 & R & -R\\ -R & 0 & R \end{array}\right]\left[\begin{array}{c} i_{A}\\ i_{B}\\ i_{C} \end{array}\right]+\left[\begin{array}{ccc} L-M & M-L & 0\\ 0 & L-M & M-L\\ M-L & 0 & L-M \end{array}\right]\frac{d}{dt}\left[\begin{array}{c} i_{A}\\ i_{B}\\ i_{C} \end{array}\right]+\left[\begin{array}{c} e_{A}\\ e_{B}\\ e_{C} \end{array}\right]$$

Electromagnetic torque:

(49) $$T_{e}=\frac{e_{A}i_{A}+e_{B}i_{B}+e_{C}i_{C}}{\Omega }$$

Motor motion equation:

(50) $$T_{e}-T_{L}=J\frac{d\omega }{dt}+B_{v}\omega$$

where $T_{L}$ is the load torque, J is the rotor rotational inertia, and $B_{v}$ is the viscous friction coefficient.

To control the brushless DC motor, it is necessary to establish the state equation of the brushless DC motor, which can be obtained by algebraic transformation of the differential mathematical model.

### 5.3. Design of Wrist Joint Based on Position PID Control

The joint motor of the manipulator adopts position closed-loop control to form a feedback loop in the control chain to reduce the accumulated error of the manipulator during the movement process and improve the accuracy of the manipulator joint tracking. The encoder feeds back the joint angle information to the controller, feeds back the actual current of the current motor through the current sensor, and adds speed feedforward based on the position control. On the premise of ensuring the accuracy of the joint control of the manipulator, it can improve the dynamic response ability of the joint and make the joint of the manipulator more flexible and smoother in the process of rotation.

The robot arm joint motor uses position closing control to form a feedback loop in the control chain. The classical PID controller [27] can reduce the additional error caused by the robot arm in the process of motion, and improve the accuracy of robot arm joint tracking. Among them, the digital increment PID controller can improve the reliability of the system, and the calculation amount of the algorithm is small. The algorithm is used as the joint control strategy of the manipulator, as shown in Formula (51)

(51) $$u(k)=\;K_{P}\left[e(k)+\frac{T}{T_{I}}\sum_{j=0}^{k}e(j)+\frac{TD}{T}(e(k)-e(k-1))\right]$$

(52) $$\begin{array}{l} △u\left(k\right)\;\;=\;\;u\left(k\right)-u\left(k-1\right)\\ \;\;\;\;\;=\;\;K_{P}(e(k)-e(k-1))+K_{I}e(k)+K_{D}(e(k)-\\ \;\;\;\;2e(k-1)+e(k-2)) \end{array}$$

In Equations (51) and (52), $K_{P}$ is the proportional factor, $K_{I}$ represents the integration factor; $K_{D}$ is the division factor. T represents the sampling period; e(k), e(k−1) represents the input error of k, k−1 sampling time. During the experiment, the changes in the three parameters $K_{P}$, $K_{I}$, and $K_{D}$ of the PID will affect the deviation between the actual value and the expected value, as well as the time when the actual value approaches the expected value. $K_{P}$ is used to accelerate system response speed and improve system adjustment accuracy. When there is a deviation between the actual value and the expected value, the regulator immediately amplifies the deviation output; $K_{I}$ is used to eliminate steady-state errors. If there is a deviation between the actual value and the expected value, even if the deviation may be small, the longer it exists, the larger the output signal. When the deviation is eliminated, the output stops changing; $K_{D}$ is used to improve the steady-state performance of the system. The larger the $K_{D}$ adjust, the better the system can respond in advance, but it will also amplify unnecessary deviations [28,29]. The PID control parameter tuning method tuning steps are first proportional, then integral, then differential. First, adjust the proportional parameters, reduce the proportional value by 10–20%, gradually add the integration time from large to small, until a 4:1 attenuation process is obtained. After adjusting the proportion and integration parameters, increase the proportion value by 10–20%, and then gradually add the differential time from small to large. Watch the transition curve until a satisfactory transition process is obtained.

**Figure 13:**
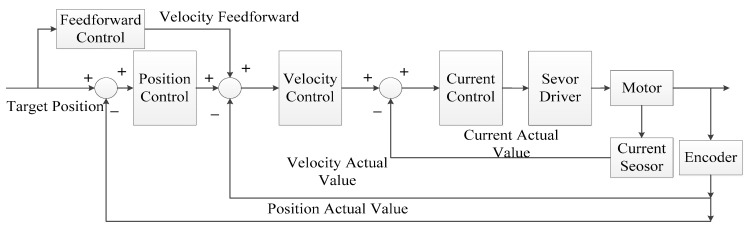
Control block diagram of wrist joint motor.

The encoder feeds back the joint angle and angular velocity information to the position loop and the speed loop, respectively. The current loop uses the current sensor to feedback on the actual current of the current motor. In the DC brushless motor control, the most commonly used is position control [30], in which the manipulator tracks the required position through control commands. This method is very accurate in a stable state and can ensure the accuracy of repeated positioning, but the dynamic performance of individual position control is poor [31]. Feedforward control [32] can compensate for disturbance and has good momentum performance. Therefore, this paper adds speed feedforward control based on position control. The general block diagram of wrist joint motor control is shown in Figure 13.

(1) Current loop PI control

**Figure 14:**
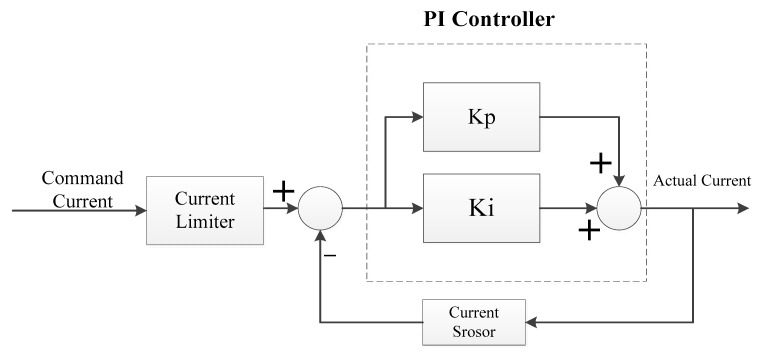
Block diagram of the current loop.

The current loop adopts a PI controller [33], namely proportional and integral regulation, to limit the maximum current of the motor, regulate the dynamic structure of the object, and speed up the dynamic response of the system. The block diagram of the current loop is shown in Figure 14.

(2) Speed loop PI control

**Figure 15:**
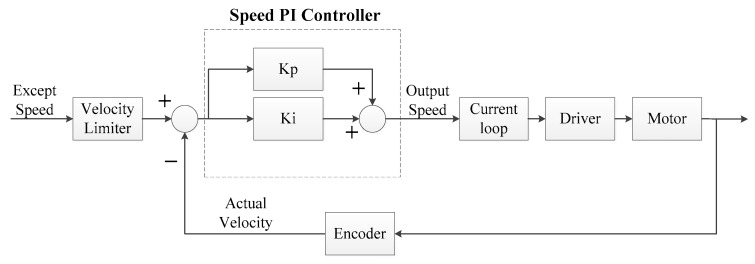
Speed loop block diagram.

The function of the speed loop is that the actual speed changes with the expected value to achieve no static error. The system has a certain anti-interference ability through closed-loop control, as shown in Figure 15.

(3) Position loop control P and Feedforward control C

**Figure 16:**
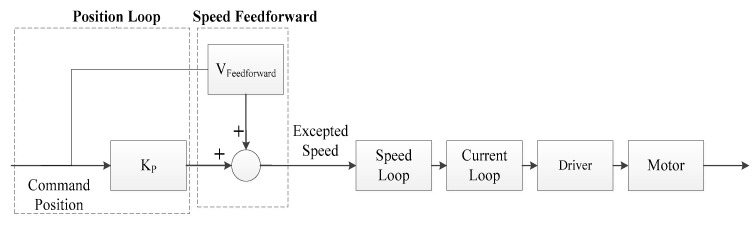
Position ring block diagram.

After the speed loop and current loop are adjusted, the desired speed can be obtained by differentiating the given positioning device. The position loop can track the given position using a proportional (P) controller, but the proportional control will have tracking lag. Based on position control, speed feedforward control is added to improve the response of the joint control system and reduce the position and speed errors. As shown in Figure 16. The simulation parameters of the system are shown in Table 5.

### 5.4. Simulation of the Established Dynamic Model

According to the wrist joint position control block diagram and the differential equation of the DC brushless motor, the Simulink mathematical simulation model is established. This paper uses the Simscape module of Simulink, which is very intuitive for modeling and simulation analysis of the wrist joint motion system. To verify the correctness of the designed controller parameters, the simulation conditions are set as follows: the initial position is 0 degrees, the expected position is 10 degrees, the initial load torque $T_{L}$ is 0 N·m, and the load torque is 5 N·m, when it is equal to 0.2 s.

**Figure 17:**
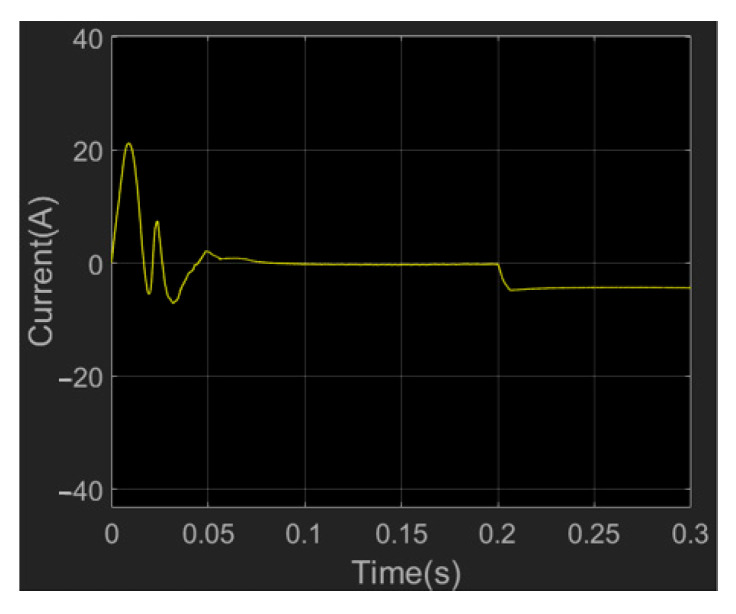
Current simulation diagram of wrist joint motor.

As shown in Figure 17, the motor will generate a large current when starting, and the system will reach a stable state in about 0.08 s. In 0.2 s, when 5 N·m load torque is applied, the current loop quickly reaches the expected current and remains stable, which verifies that the current loop control meets the control requirements of the manipulator joint.

**Figure 18:**
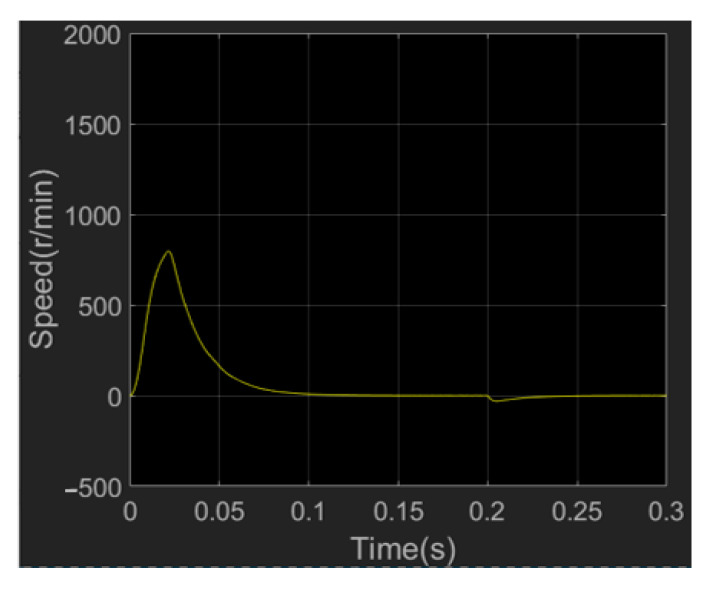
Simulation diagram of wrist joint motor speed.

According to the simulation results of the speed in Figure 18, the speed of the motor experienced two processes. The speed of the motor first increases from zero, and then decelerates to about 0.08 s, so it can quickly reach a stable state. The system has a fast dynamic response capability. After adding 5 N load in 0.2 s, the speed fluctuates slightly and returns to a stable state in a short time, which indicates that the control system is robust to variable load.

**Figure 19:**
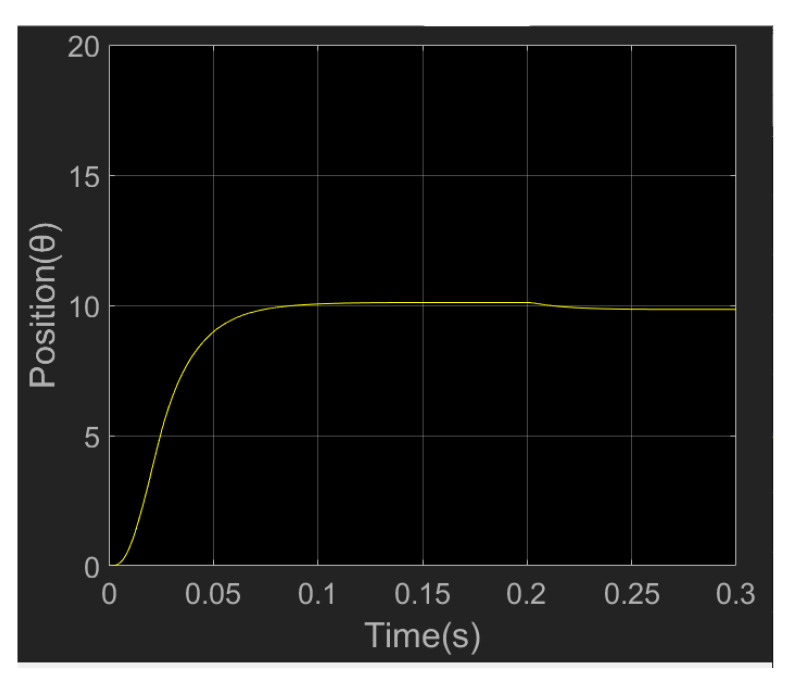
Simulation diagram of wrist joint motor position.

From the position simulation diagram in Figure 19, it can be seen that when the wrist joint motor moves from the initial position of 0 to 10 degrees, the slope of the position curve first increases and then decreases, and reaches the required position in about 0.08 s. After adding a 5 N load at 0.2 s, the position loop has a small tracking error, which can meet the needs of actual motor control performance.

According to the above simulation results, it can be verified that the feedforward and position control strategies have good dynamic performance and anti-interference ability, and can meet the accuracy requirements of wrist joint motor control.

## 6. Wrist Joint Motor Test

### Actual Test Data of Position, Speed, and Current under Different Loads

**Figure 20:**
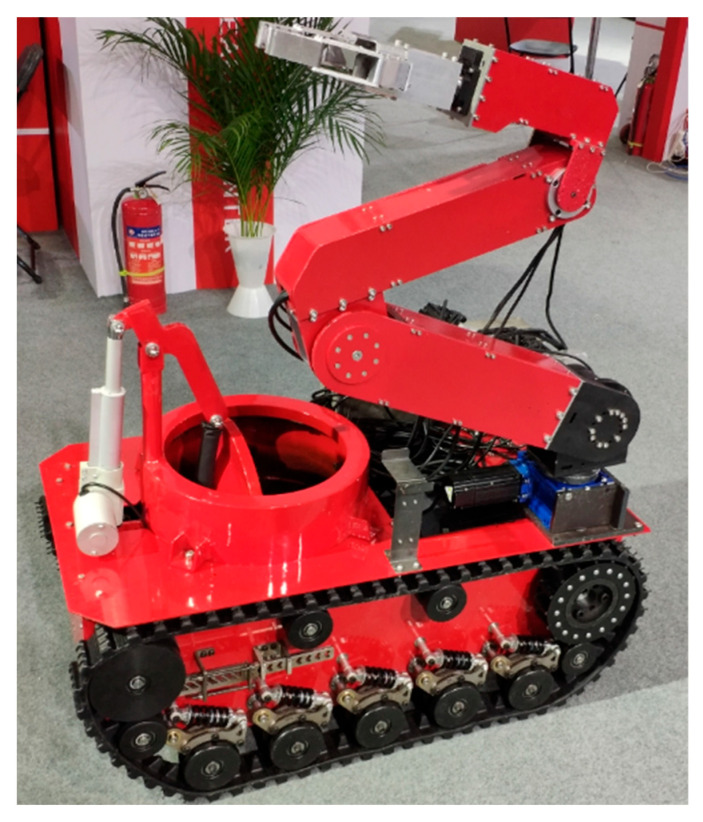
Wrist joint loading diagram.

The actual test was performed on the forearm elbow joint. This test is divided into two groups. The first group of wrist joints is not loaded. The second group of mechanical arm wrist joints increased the load by 0.5 kg, as shown in Figure 20.

The first group is the no-load test.

**Figure 21:**
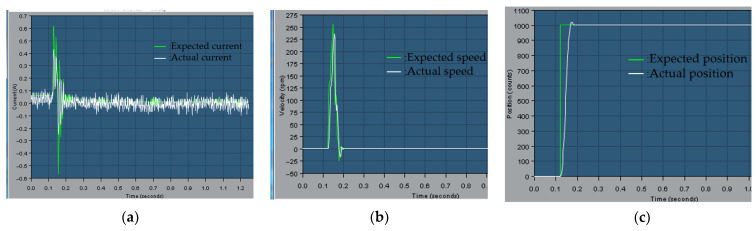
(**a**) Expected current and actual current curve of wrist joint motor; (**b**) Expected speed and actual speed curve of wrist joint motor; (**c**) Expected position and actual position curve of wrist joint movement.

Figure 21a shows the expected current and actual current curve of the wrist joint motor. The green curve represents the expected current and the white curve represents the actual current. It can be seen from the figure that the actual current can well track the expected current through feedforward control. As shown in Figure 21b, the green curve is the expected speed curve, and the white curve is the actual speed curve. Under position control, the motor accelerates first, then decelerates, reaches the maximum speed of 250 rpm in about 0.15 s, and finally remains unchanged. As shown in Figure 21c, the wrist joint motor moves from the initial position of 0 to 1000 counts. The green curve is the expected position of the wrist joint, and the white curve is the actual position of the wrist joint. It is slightly too large at the actual position of about 0.18 s. Then, we track the desired position on the actual position, and the system reaches a stable state.

From the above actual test results, it can be seen that under the feedforward control, the actual position of the wrist joint can quickly track the desired position.

The second group is the wrist joint position, speed, and current curve after adding 0.5 kg load.

**Figure 22:**
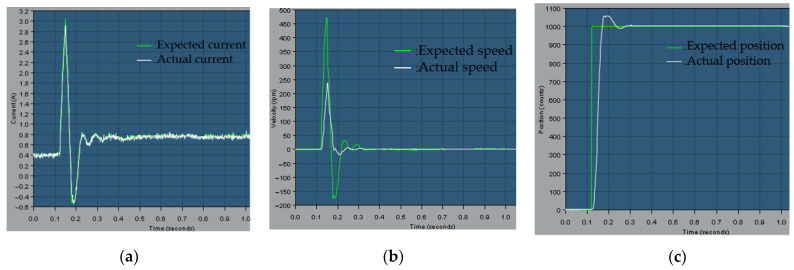
(**a**) Expected current and actual current curve of wrist joint motor; (**b**) Expected speed and actual speed curve of wrist joint motor; (**c**) Expected position and actual position curve of wrist joint movement.

As shown in Figure 22a, the expected current curve and the actual current curve after the motor is loaded. It can be seen from the figure that under the feedforward control, the actual current curve can better track the expected current curve without delay. As shown in Figure 22b, the curve between the expected speed and the actual speed of the wrist joint motor and the load is shown. When rotating at a certain angle, the actual speed of the wrist motor can quickly track the desired speed, but there will be some errors. As shown in Figure 22c, after the wrist joint bears a load of 0.5 kg, it moves back and forth. The green curve is the expected position of the wrist joint, and the white curve is the actual position of the wrist joint. In about 0.18 s, the actual position shows obvious supersimulation. After the controller is adjusted, the desired position is tracked on the actual position within 0.3 s, and the system reaches a stable state.

The wrist joint-related parameters under no-load and load conditions are organized as shown in Table 6. Table 6 analyzes the expected current and actual current of the wrist joint motor, the expected speed and actual speed of the motor, and the curves of the expected position and actual position of the wrist joint movement.

From the above actual test results, it can be seen that under the feedforward control, the wrist joint has good robustness to variable load conditions while ensuring position control accuracy. The control methods of feedforward control and position fusion not only have the fastest speed of feedforward control but also have the precise characteristics of feedback control. The controller has good dynamic response performance and anti-interference ability.

## 7. Conclusions

A robot arm with a folding manipulator is designed in this paper, and the correctness of the kinematics algorithm is verified by modeling, analysis, and simulation. A fusion method of speed feedforward and position control is proposed, which can be used to solve the control problem of manipulator joints. The closed-loop feedback adopts a PID controller to correct the error, and the speed feedforward compensates for the dynamic performance of the system. The simulation results show that the strategy has a fast dynamic response ability and can converge quickly under the premise of ensuring accuracy. The effectiveness of the simulation results is further verified by experiments. The results indicate that compared to traditional five-DOF robotic arm systems, the control system of this robotic arm has lower tracking delay and better dynamic response performance. It can improve the system response performance while also enhancing its anti-interference ability. The robot arm joint is smoother in the process of rotation and can meet the precision requirements of wrist motor control. The results show that the method can meet the operation requirements of the robot arm and provide a practical basis for realizing the trajectory motion of the robot arm in complex space.

## Figures and Tables

**Table 1:** Range of motion of manipulator joint.

Mechanical Claw	Wrist Joint	Elbow Joint of Forearm	Middle Arm Elbow	Shoulder Joint 2 (Retractable)	Shoulder Joint 1 (Rotation)
−360°~+360°	−360°~+360°	0~120°	0~180°	−30°~+210°	−180°~+180°

**Table 2:** D-H parameter table of manipulator after introducing virtual joint.

Joint	$d_{i}$	$a_{i}$	$\alpha_{i}$	$\theta_{i}$	Joint Range/°
Shoulder joint 1	$d_{1}$	0	Pi/2	$\theta_{1}$	0~180
Shoulder joint 2	0	$a_{2}$	0	$\theta_{2}$	−60~180
Middle arm elbow	0	$a_{3}$	0	$\theta_{3}$	−120~90
Virtual joint	0	$a_{4}$	0	$\theta_{4}$	90
Elbow joint of forearm	0	$a_{5}$	Pi/2	$\theta_{5}$	−90~90
Wrist joint	$d_{6}$	0	0	$\theta_{6}$	−180~180

**Table 3:** Four groups of inverse kinematics solutions.

Order Number	Joint	θ/rad	Joint	θ/rad
1	Shoulder joint 1	1.0472	Shoulder joint 2	0.9839
	Middle arm elbow	−1.0472	Virtual joint	1.5708
	Elbow joint of forearm	0.5869	Wrist joint	1.5708
2	Shoulder joint 1	1.0472	Shoulder joint 2	1.0472
	Middle arm elbow	−1.0472	Virtual joint	1.5708
	Elbow joint of forearm	0.5236	Wrist joint	1.5708
3	Shoulder joint 1	1.0472	Shoulder joint 2	1.1038
	Middle arm elbow	−1.1671	Virtual joint	1.5708
	Elbow joint of forearm	0.5869	Wrist joint	1.5708
4	Shoulder joint 1	1.0472	Shoulder joint 2	1.1671
	Middle arm elbow	−1.1671	Virtual joint	1.5708
	Elbow joint of forearm	0.5236	Wrist joint	1.5708

**Table 4:** The relevant parameters and interface functions of the controller.

Basic Parameters	Numerical or Interface Functions
MCU	STM32F103
Communication protocol	CAN bus protocol
Baud rate	500 K Bit/s
Sampling time point	1.75 μs
CAN interface	Used for communication of functional testing
JTAG interface	Used for DAP online simulation and program download

**Table 5:** Simulation parameters.

Basic Parameters	Numerical Value
Proportional gain $K_{p}$	200
Integral term $K_{I}$	3.3
Differential term $K_{D}$	2.2
Simulation time T	0.3 s

**Table 6:** Experimental results of current, speed, and position under no-load and loaded conditions.

Wrist Joint	Motor Current	Motor Speed	Wrist Joint Position
No load	During the period of 0.12 s to 0.19 s, the expected current rises from 0.07 A to 0.62 A and then changes to −0.58 A, then fluctuates within the range of −0.1 to 0.1 A. After detecting significant fluctuations in the expected current within 0.12 s, corresponding adjustments were made, and the overall trend of change is the same as the expected current	During the period from 0.12 s to 01.19 s, the expected speed of the wrist joint motor increased from 0 to 258 rpm, then decreased to −25 rpm, and stabilized around 0 in 0.2 s. During the period from 0.12 s to 01.19 s, the actual speed increased from 0 to 238 rpm and then decreased to −17 rpm, with a delay of about 0.01 s compared to the expected speed curve.	At 0.12 s, the expected position of wrist joint movement suddenly changed from 0 to 1000, and the actual movement position continued to rise between 0.12 s and 0.18 s, stabilizing around 1000 around 0.19 s.
With load	During the period from 0.12 s to 0.19 s, there was a significant change in the expected current, rising from 0.43 A to 3.03 A and then dropping to −0.56 A. After 0.33 s, there was a slight fluctuation around 0.76 A; During the period from 0.12 s to 0.19 s, the actual current increased from 0.42 A to 2.95 A, then decreased to −0.54 A, and fluctuated slightly around 0.75 A after 0.32 s. The actual current curve can closely track the expected current curve without delay, and the waveforms of the two are close to overlapping	During the period from 0.12 s to 0.3 s, the wrist joint rotated at a certain angle, and the expected speed of the motor changed from 0 to 475 rpm, then decreased to −175 rpm, and stabilized around 0 after 0.32 s. During the period of 0.12 s to 0.3 s, the actual speed of the motor increased from 0 to 240 rpm and then decreased to −20 rpm, stabilizing around 0 after 0.25 s.	At 0.12 s, the expected position of wrist joint movement suddenly changed from 0 to 1000, and the actual motion position continued to rise during the period of 0.12 s to 0.18 s. Around 0.18 s, the actual position showed supersimulation, and the expected position was tracked at 0.3 s.

## Data Availability

The data are available upon request.
